# Effects of Tai Chi on balance and fall prevention in healthy older adults: a randomized controlled meta-analysis

**DOI:** 10.3389/fpubh.2025.1638006

**Published:** 2026-01-06

**Authors:** Runqiang Dong, Mohd Sahandri Gani Bin Hamzah, Mohd Mahzan Bin Awang, Jianguo Qiu, Shufang Chen

**Affiliations:** 1 Faculty of Education, Universiti Kebangsaan Malaysia, Bangi, Selangor Darul Ehsan, Malaysia; 2 School of Physical Education, Ludong University, Yantai, Shandong, China; 3 School of Physical Education, Pingxiang University, Pingxiang, Jiangxi, China

**Keywords:** Tai Chi, balance, fall prevention, healthy older adults, meta-analysis

## Abstract

**Objective:**

This study aimed to evaluate the effects of Tai Chi on balance and fall risk in healthy older adults through a systematic review and meta-analysis of randomized controlled trials (RCTs).

**Methods:**

A comprehensive literature search was conducted across international and Chinese databases to identify relevant studies published between 2004 and 2024. A total of 21 RCTs were included in the analysis, comparing Tai Chi with non-exercise control groups. Various balance measures, such as balance performance and fall risk, were assessed.

**Results:**

The meta-analysis revealed that Tai Chi significantly improved balance and reduced fall risk among older adults. Specifically, improvements were observed in several key measures, including balance ability, walking speed, and confidence in preventing falls.

**Conclusion:**

These findings suggest that Tai Chi is an effective intervention for enhancing balance and reducing fall risk in healthy older adults, highlighting its potential as a valuable preventive strategy for falls in the aging population.

**Systematic review registration:**

https://www.crd.york.ac.uk/prospero/, identifier CRD420251004905.

## 1 Introduction

The global trend of population aging is posing an urgent public health challenge. Projections indicate that by the mid-21st century, approximately 20% of the world’s population will be 60 years or older (1). As age increases, the human body inevitably undergoes physiological changes. Even among older adults who are considered healthy, balance ability may decline with aging (2). Due to the deterioration of balance ability and proprioception, the gradual degeneration of dendrites, and the age-related decline in neurotransmitters such as dopamine, acetylcholine, and serotonin, the signal transmission in the postural control center decreases or becomes abnormal. This leads to a slower information processing speed, reducing the ability of older adults to respond to various situations and further increasing the risk of falls (3–5). According to relevant data, approximately 6% of older adults sustain injuries due to falls each year, with around 1% of these incidents resulting in hip fractures (6). The 2021 World Health Organization (WHO) report stated that falls are the second most common cause of injury-related deaths among older adults. The report highlights a significant increase not only in the incidence of falls but also in the associated mortality rate, with individuals aged 60 years and older being the most affected (7). Falls have become a major global health concern for older adults and are the leading cause of accidental injuries and deaths in this population (8). Since 1990, the total number of deaths and disability-adjusted life years (DALYs) lost due to falls has been continuously increasing. According to the Global Burden of Disease (GBD) Report, falls resulted in approximately 17 million years of life lost in 2017 alone (9). The associated social and economic consequences are extremely severe, highlighting the urgent need for the development of targeted prevention and intervention strategies.

Numerous studies have shown that regular exercise can significantly slow down the decline in muscle function during the aging process (10–12). Relevant studies have indicated that exercise interventions play a significant role in improving balance and preventing falls in older adults. Such interventions can induce a series of beneficial muscular adaptations, including increased aerobic enzyme activity, enhanced muscle contractility, and significant improvements in muscle strength and activation (12). Moreover, exercise has been proven to enhance the sensitivity of ligaments, joint capsules, and muscle receptors, thereby improving neuromuscular control and accelerating sensory input transmission. By enhancing the integration of sensory information, exercise interventions can effectively improve postural control in older adults while strengthening the overall function of the sensorimotor system (13).

Tai Chi, also known as Tai Chi Chuan, was created by the renowned Chinese martial artist Chen Wangting during the late Ming Dynasty. It is a balance-based exercise guided by the traditional Chinese medicine (TCM) theory of Yin and Yang (14). Tai Chi Chuan integrates elements of traditional Chinese folk martial arts, military combat techniques, breathing and meditation practices, as well as the core principles of Yin-Yang philosophy, forming a unique mind–body cultivation system (15). The unique characteristics of Tai Chi Chuan, including controlled displacement of body mass over the base of support, postural orientation, range of motion (e.g., ankle and hip movements), and emphasis on abdominal and lower limb muscle function, may contribute to greater postural stability through enhanced biomechanical mechanisms. These factors help improve balance function in older adults, reduce the likelihood of falls, and lower the risk of fall-related injuries (16–18). To date, numerous clinical trials (19–21) and systematic reviews (22–25) have been conducted to evaluate the effectiveness of Tai Chi in improving balance and reducing fall risk among older adults. However, the findings remain inconsistent, with some studies reporting significant benefits of Tai Chi (19, 20), However, other studies have failed to confirm its effectiveness. For example, a study by Logghe et al. (26) demonstrated that Tai Chi did not show significant effects on improving balance or preventing falls in older adults. The study concluded that the current evidence is insufficient to support the effectiveness Moreover, most studies have primarily focused on specific subgroups with health impairments, such as individuals with sarcopenia or Parkinson’s disease, while research on healthy older adults remains relatively scarce. In particular, meta-analyses systematically evaluating the effects of Tai Chi on different aspects of balance have not been sufficiently conducted. Therefore, this study aims to perform a meta-analysis to synthesize the available evidence and assess the weighted mean difference (WMD) of Tai Chi’s impact on balance ability and fall prevention in healthy older adults. Additionally, it explores the quantitative effects of Tai Chi style, training frequency, and intervention duration on these parameters. The findings will provide a scientific basis for designing evidence-based intervention programs and further enhancing the well-being and quality of life of older adults.

Tai Chi is a slow, cyclic movement practice that is often described as involving alternating phases of focus and release (27). This alternating focus may help improve motor control and stability, particularly in older adults. The practice encourages attentional engagement during movement and provides restorative phases where attention is allowed to shift and relax. This balance between attentional focus and restoration can contribute to enhanced proprioception and postural control. According to Schumann et al., the continuous, slow movements of Tai Chi align with attentional engagement and restoration theory, which suggests that sustained focus on controlled movement helps stabilize cognitive resources and improves motor control (28). Additionally, Tai Chi movements create a low physiological arousal state, as described by Steghaus and Poth, which can promote physical relaxation while maintaining mental focus. This low-arousal state is key for improving balance and posture in older adults, as it supports relaxation without compromising stability (29). Furthermore, the benefits of Tai Chi in balancing cognitive and motor processes are consistent with Thayer’s (1989) biopsychology of mood and arousal, which links physiological arousal to emotional and motor readiness (30). Together, these theories help explain why Tai Chi is an effective intervention for maintaining balance and preventing falls in older adults.

While previous reviews have investigated the effects of Tai Chi on older adults, many of these studies have either included participants with various health conditions or have focused on specific aspects of balance without considering the influence of training parameters such as duration, frequency, and style (31, 32). There is limited evidence on the effectiveness of Tai Chi specifically for healthy older adults, and even less is known about the optimal dose (e.g., frequency and session length) and Tai Chi styles for balance improvement and fall prevention. This meta-analysis aims to fill this gap by focusing on healthy older adults and examining how different intervention parameters, including Tai Chi style and training duration, influence balance and fall risk. By synthesizing existing studies, this work seeks to provide a clearer understanding of the role of Tai Chi in fall prevention and balance enhancement for older adults, with a particular focus on identifying effective intervention dosages and styles.

This study systematically searched international and Chinese databases and included randomized controlled trials (RCTs) published between 2004 and 2024, with a total of 21 studies meeting the inclusion criteria. These studies compared Tai Chi with non-exercise control groups. The interventions varied in terms of duration, weekly frequency, and specific Tai Chi styles (e.g., Yang-style 24-form Tai Chi, Simplified 24-form Tai Chi, Chen-style Tai Chi, etc.). Data on standard measures related to balance performance, stability during standing and movement, walking speed, and confidence in maintaining balance were extracted. Meta-analytic procedures were used to estimate pooled effects, and subgroup analyses were conducted to explore whether training duration, session length, frequency, or style influenced outcomes. The results indicate that Tai Chi significantly improves several balance-related measures and may contribute to a reduction in fall risk. However, substantial heterogeneity among studies and potential biases in research designs may affect the consistency and reliability of the findings. Future studies should standardize intervention protocols and outcome measurement tools to enhance comparability.

## 2 Research methods

This systematic review and meta-analysis strictly adhered to the PRISMA statement and PERSIST guidelines (33, 34), The entire review process has been formally documented and registered in the PROSPERO database under the registration number CRD420251004905.

### 2.1 Inclusion criteria

The inclusion criteria for this meta-analysis were strictly defined to ensure the highest methodological quality. Studies were required to be randomized controlled trials (RCTs) published between 2004 and 2024, comparing Tai Chi interventions with non-exercise control groups. Only studies involving healthy older adults, aged 60 and above, were included. Studies that did not report relevant balance-related outcomes or lacked full-text availability were excluded.

#### 2.1.1 Research population

Participants included older adults aged 60 years and above who had not participated in or practiced Tai Chi in the past 6 months, regardless of nationality or ethnicity. Individuals with severe acute or chronic diseases or those unable to comply with the study protocol were excluded.

#### 2.1.2 Intervention

The intervention group participated in Tai Chi practice, while the control group received no form of exercise intervention and maintained their usual lifestyle and daily activities.

#### 2.1.3 Outcome measures

The primary outcome measures of this study included the assessment of balance ability and fall risk, evaluated using the Fear of Falling (FOF) test, Falls Efficacy Scale (FES), Timed Up and Go (TUG) test, Time Balance Test (TBT), functional reach distance (FRD), maximum walking speed (MWS) test, Berg Balance Scale (BBS), and One-Leg Standing Test (OLS-C) (as shown in Table 1).

**Table 1:** Outcome measures and descriptions.

Outcome measure	Full Name	Description
TUG	Timed Up and Go Test	A performance-based measure assessing mobility, balance, and functional ability. It measures the time taken for a participant to stand up from a chair, walk 3 meters, turn around, return, and sit down.
BBS	Berg Balance Scale	A performance-based scale assessing balance in older adults, using tasks such as standing up from a chair, reaching, and turning. It evaluates functional balance in various positions.
FRD	Functional reach distance	A performance-based measure evaluating balance and postural control by assessing how far a person can reach forward without losing balance.
FOF	Fear of Falling	A self-reported measure assessing an individual’s fear of falling. It uses a scale where individuals rate their fear of falling during different activities.
FES	Falls Efficacy Scale	A self-reported measure of an individual’s confidence in their ability to perform various activities without falling. It captures psychological perceptions of stability.
TBT	Timed Balance Test	A performance-based test used to evaluate balance and postural control by assessing the time taken to complete certain tasks like standing on one leg.
MWS	Maximum Walking Speed	A performance-based measure of walking ability, assessing the maximum speed at which a participant can walk a certain distance. It provides insight into dynamic balance and mobility.

#### 2.1.4 Research design

Systematic review with meta-analysis.

#### 2.1.5 Control condition

In the included studies, the control condition was defined as “no exercise,” but it is important to clarify that this can vary across studies. In some studies, the control group simply continued with their usual daily activities, which may or may not involve physical exercise. In other studies, the control group was asked to engage in a structured waiting period with no physical activity, while some studies involved a light health education session, focusing on general health advice without physical exercise. These control conditions were chosen to ensure that any observed effects could be attributed to Tai Chi rather than other forms of physical activity. By defining the control groups in these ways, we aimed to isolate the specific effects of Tai Chi on balance and fall prevention in healthy older adults.

### 2.2 Exclusion criteria

Several studies were excluded based on specific criteria to ensure the quality and focus of this analysis. Studies involving individuals with severe acute or chronic diseases (e.g., Parkinson’s disease, osteoporosis, or other serious health conditions) were excluded. Additionally, studies that combined Tai Chi with other interventions, such as strength training or aerobic exercises, were not included, as the aim of this meta-analysis was to evaluate the standalone effect of Tai Chi. Furthermore, studies with non-standardized interventions, such as varying Tai Chi styles or training durations, were excluded due to methodological heterogeneity. This was done to ensure that the results could be interpreted with greater consistency and clarity.

While studies that combined Tai Chi with other forms of activity (e.g., strength training, aerobic exercise) were excluded, this decision was based on the judgment that the effects of Tai Chi could not be isolated from the effects of other interventions in those studies. However, it is important to note that some combined interventions may have placed Tai Chi as the dominant component of the exercise regimen, and thus could have had a primary impact on balance and fall risk. The exclusion of these studies was not solely based on automated filtering via search terms, but was carefully evaluated on a case-by-case basis to ensure that the analysis focused on the isolated effect of Tai Chi. This process of evaluation allowed for a clearer understanding of Tai Chi’s specific role in improving balance and reducing fall risk in older adults.

#### 2.2.1 Duplicate publications

This study will exclude duplicate publications of previous research to avoid redundancy.

#### 2.2.2 Articles with inaccessible full text will be excluded

Since full-text review is essential for assessing study quality, articles with inaccessible full text will be excluded.

#### 2.2.3 Articles with incomplete data will be excluded

Studies with insufficient data or incomplete datasets that cannot be used for meta-analysis will be excluded.

#### 2.2.4 Studies published in non-core Chinese journals or non-SCI-indexed English journals will be excluded

To ensure that the included studies strictly adhere to peer-review standards and to minimize the bias introduced by low-quality research, studies published in non-core Chinese journals and non-SCI-indexed English journals will be excluded. This measure aims to enhance the accuracy and reliability of the study findings.

#### 2.2.5 Studies in which the intervention is not solely Tai Chi will be excluded

Studies in which the intervention group does not exclusively practice Tai Chi will be excluded. This includes studies where Tai Chi is not the primary or sole intervention or is combined with other treatment methods, as such variations may affect the accuracy of the results.

### 2.3 Search strategy

This study conducted a comprehensive literature search in China National Knowledge Infrastructure (CNKI), PubMed, Web of Science, Cochrane Library, Embase, Google Scholar, and Wan fang Database. The search covered studies published between 2004 and 2024, encompassing nearly 20 years of relevant research. The search strategy combined Medical Subject Headings (MeSH) terms and free-text keywords to ensure a comprehensive and systematic retrieval of eligible studies. The Chinese search terms were:(老年人或老人)和(太极或太极拳或太极运动)和(平衡或平衡能力或跌倒). The English search terms were: (Aged OR Aging OR Older adults OR Senior) AND (“Tai Ji” OR “Tai-ji” OR “Tai Chi” OR “Tai Ji Quan” OR “Taijiquan” OR “T’ai Chi”) AND (“Balance” OR “Falls” OR “Falling” OR “Fall Risk”). AND (“Randomized Controlled Trials as Topic” OR “Clinical Trials Randomized” OR “Trials”). The search terms were adjusted according to the specific requirements of each database. For example, in PubMed, the detailed search strategy is presented in Table 2.

**Table 2:** PubMed search strategy.

Search strategy
#1” Tai Chi” [Mesh]
#2″ T’ai Chi”OR” Tai Ji Quan”OR” Tai Ji”OR” Ji Quan, Tai”OR” Quan, Tai Ji”[All files]
#3#1OR#2
#4”Aged”[Mesh]
#5” Aging”OR” Older Adult”OR” Elderly”OR” older adults”OR” Older Adult”OR” senior” [All files]
#6#4OR#5
#7”Accidental Falls”[Mesh]
#8” Falls”OR” Falling”OR” Fall Risk”OR” Balance” [All files]
#9#7OR#8
#10” Random” [Mesh]
#11”Randomized Controlled Trials as Topic “OR” randomized”OR” placebo” [All files]
#12#10OR#11
#13#3AND#6AND#9AND#12

### 2.4 Quality assessment of included studies

In this study, two researchers independently assessed the quality of the included studies based on the *Cochrane Handbook for Systematic Reviews of Interventions (Version 5.10)*. The specific assessment criteria included selection bias (random sequence generation and allocation concealment), performance bias (blinding of participants and intervention providers), detection bias (blinding of outcome assessors), attrition bias (completeness of outcome data), reporting bias (selective outcome reporting), and other potential sources of bias. The evaluation results were categorized into three levels: studies that fully met the above criteria were classified as Grade A; those that partially met the criteria were classified as Grade B; and those that did not meet the criteria were classified as Grade C. In cases where discrepancies arose between the two researchers, a third-party reviewer was consulted for discussion or adjudication until a consensus was reached.

### 2.5 Data extraction

Two researchers will independently extract data and rigorously review the relevant literature based on predefined inclusion and exclusion criteria. To ensure the systematic and consistent collection of data, this study will use a standardized data extraction form, which will primarily collect the following key information:

(1) Basic Information: Including the first author of the study, year of publication, and the mean age and standard deviation (M ± SD) of the participants.
(2) Sample Size: The total number of participants in each study.
(3) Study Design and Intervention: A comprehensive record of the study design and detailed description of the intervention, including the duration of the intervention, frequency of sessions, and specific outcome measures used.

During the data extraction process, any discrepancies will be resolved through discussion among the researchers until a consensus is reached. For studies with incomplete reports, the original authors will be contacted via email to obtain the missing data. If the required information cannot be obtained, the study will be excluded from the final analysis.

### 2.6 Data analysis

Data analysis was conducted using Review Manager 5.4 and Stata SE V.15 software. A random-effects model or fixed-effects model was applied to calculate the weighted mean difference (WMD) and 95% confidence interval (CI) for the impact of Tai Chi on various balance-related outcome measures in older adults. The analysis was performed based on the reported mean and standard deviation (M ± SD) from each study. The direction of WMD was determined by the specific outcome measure used (35). Heterogeneity of the studies was assessed using the I^2^ statistic. If *I*^2^ was ≤50%, the heterogeneity was considered acceptable (36). When overlapping confidence intervals and heterogeneity were observed, the corresponding *p*-value was analyzed. If the heterogeneity test result was not significant (*I*^2^ < 50% or *p* > 0.1), the included studies were considered homogeneous, and the Mantel–Haenszel fixed-effects model was applied (37). If heterogeneity was significant (*I*^2^ > 50% or *p* ≤ 0.1), the potential sources of heterogeneity were first analyzed, considering factors such as measurement methods, intervention protocols, gender, age, and control group selection. Heterogeneity caused by these factors was addressed using subgroup analysis to perform pooled statistical calculations. If heterogeneity remained among similar studies, a random-effects model was applied to compute the pooled effect size (38). Additionally, potential publication bias was assessed using funnel plots and Egger’s test to ensure the reliability of the conclusions.

## 3 Results

### 3.1 Characteristics of included studies

**Figure 1:**
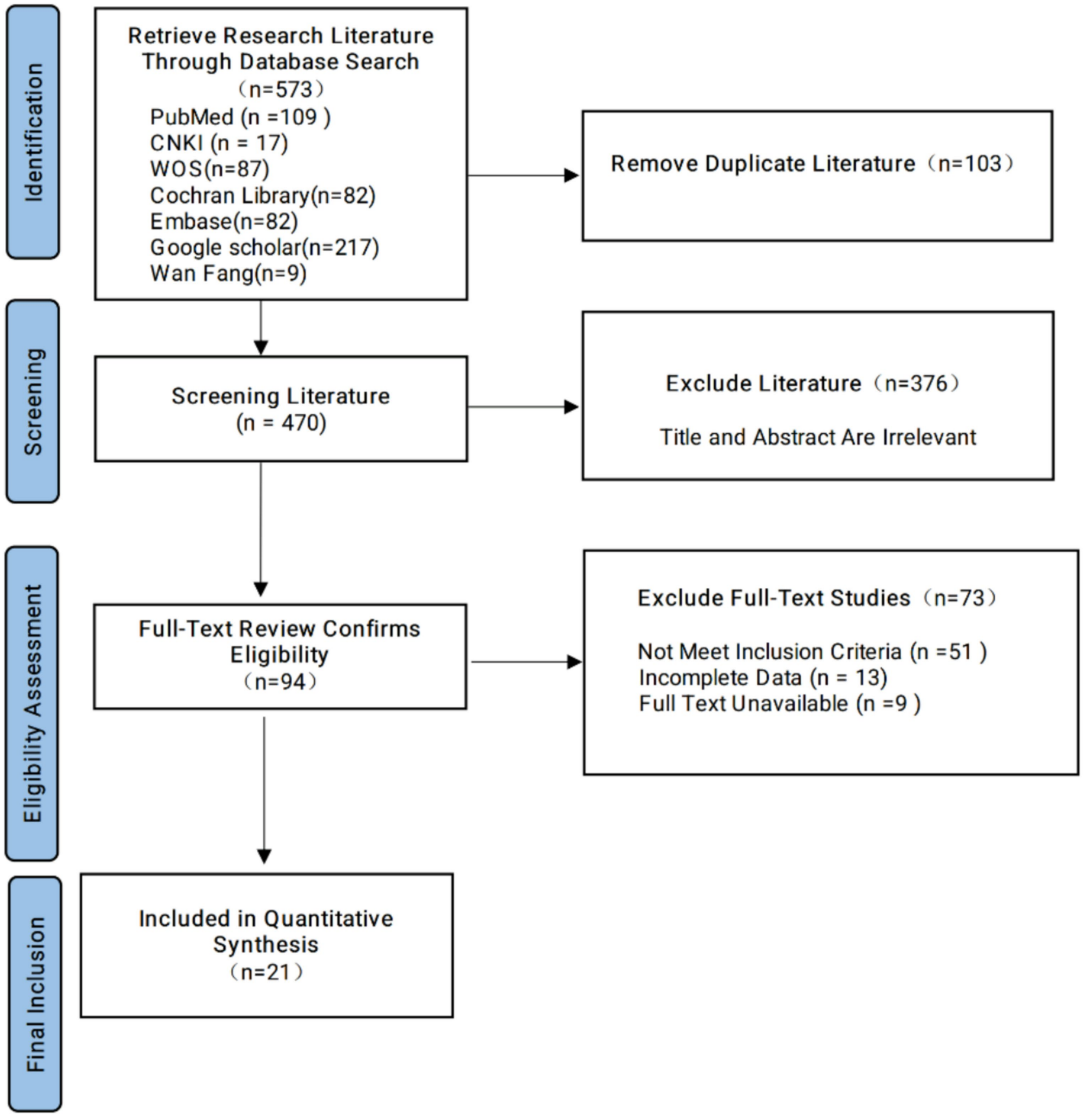
Literature search results and process.

The literature screening process and results are presented in Figure 1. A comprehensive search across multiple databases initially identified 573 studies. After removing 103 duplicate records, 470 studies remained for further screening based on title and abstract, leading to the exclusion of 376 studies that were not relevant to the research topic. The full texts of the remaining 94 studies were then assessed for eligibility. Following full-text review, 73 studies were excluded, including 51 studies that did not meet the inclusion criteria, 13 studies with incomplete data, and 9 studies that were excluded due to inaccessible full text. A total of 94 studies were identified as meeting the inclusion criteria. After quality assessment, 21 studies were included in the quantitative synthesis (meta-analysis).

Among the 21 included studies, five were published in Chinese journals (17, 39–42),16 studies were published in English journals (19, 20, 43–56), A total of 2,408 participants were included across all studies. The mean age of participants in the control group ranged from 60.3 to 70.9 years, while the mean age of participants in the intervention group ranged from 66.1 to 75.3 years. The interventions included various styles of Tai Chi, 8 studies utilized Yang-style Tai Chi (17, 19, 20, 43, 44, 48, 49, 52), 5 studies utilized the Simplified 24-Form Tai Chi (39, 40, 50, 54, 56), The remaining studies employed other styles of Tai Chi. The intervention duration in these studies ranged from 4 to 26 weeks, with two studies extending beyond 26 weeks (39, 41), The exercise frequency ranged from 2 to 4 sessions per week, with each session lasting between 30 and 90 min. The Fear of Falling (FOF) assessment was included in four studies (44, 45, 51, 56), The Falls Efficacy Scale (FES) was assessed in five studies (17, 20, 49, 50, 52), The Time Balance Test (TBT) was assessed in three studies (39–41), The Timed Up and Go (TUG) test was assessed in 12 studies (17, 19, 20, 40, 41, 45–48, 51–53), The functional reach distance (FRD) test was assessed in four studies (19, 40, 41, 49), The maximum walking speed (MWS) test was assessed in four studies (42, 47, 48, 56), The Berg Balance Scale (BBS) was assessed in five studies (17, 48, 49, 53, 55), The One-Leg Standing Test (OLS-C) was assessed in 12 studies (17, 39–41, 43, 46, 47, 51–54, 56). As shown in Table 3.

**Table 3:** Basic characteristics of included studies.

First author/year	Age	Sample size		Intervention measures style	Exercise details			Outcome measure
		Intervention group	Control group		Duration Per session	Frequency Per week	Duration	
Jiao Sun 2015 (43)	70.1 ± 5.7	66	72	Yang-style 24-Form Tai Chi	60 min	2 times	26 weeks	⑧
I-Wen Penn 2019 (19)	73.4 ± 8.2	15	15	Yang-style 24-Form Tai Chi	30 min	3 times	8 weeks	④⑦⑤
Reema Joshi 2024 (46)	66.4 ± 4.1	20	20	Chen-style Tai Chi	35 min	4 times	4 weeks	④⑧
ShuWan Chang 2014 (39)	60.3 ± 2.9	26	19	Simplified 24-Form Tai Chi	90 min	2 times	>26 weeks	③⑧
Fuzhong Li 2005 (17)	77.9 ± 5.1	131	125	Yang-style 24-Form Tai Chi	90 min	3 times	26 weeks	②⑤④⑦⑧
Toni Rikkonen 2023 (51)	76.6 ± 3.2	457	457	Traditional Tai Chi	60 min	1 time	26 weeks	①④⑧
Yan Ma 2024 (47)	64.4 ± 7.4	29	31	Hybrid Tai Chi	60 min	2 times	26 weeks	④⑥⑧
YuYu Qiu 2008 (40)	67.4 ± 8.1	26	27	Simplified 24-Form Tai Chi	40 min	7 times	10 weeks	③④⑤⑧
ChunMei Xiao 2006 (41)	67.9 ± 4.2	51	21	Conventional Tai Chi	60 min	4 times	>26 weeks	③④⑤⑧
ChunZheng Peng 2023 (42)	63.1 ± 3.8	43	45	Water-Based Perturbation Tai Chi	50 min	6 times	24 weeks	⑥
Lida Hosseini 2018 (20)	70.3 ± 5.5	30	30	Yang-style 24-Form Tai Chi	55 min	2 times	8 weeks	②④
Hamed Mortazavi 2018 (49)	68.1 ± 5.2	26	27	Yang-style 24-Form Tai Chi	20 min	3 times	10 weeks	②⑤⑦
Jian-Guo Zhang 2006 (58)	70.6 ± 4.9	23	24	Simplified 24-Form Tai Chi	60 min	7 times	8 weeks	①⑥⑧
Yujie Ge 2022 (44)	72.9 ± 6.6	33	32	Yang-style 24-Form Tai Chi	60 min	7 times	8 weeks	①
Hui-Chi Huang 2010 (45)	71.50 ± 1.3	47	31	13-Form Tai Chi	40 min	3 times	22 weeks	①④
Brad Manor 2014 (48)	86.1 ± 6.6	28	29	Yang-style 24-Form Tai Chi	60 min	2 times	12 weeks	④⑥⑦
Farzaneh Sadeghian 2023 (52)	65.1 ± 4.1	18	18	Yang-style 24-Form Tai Chi	60 min	3 times	8 weeks	②④⑧
Manh Hung Nguyen 2012 (50)	68.7 ± 4.9	48	48	Simplified 24-Form Tai Chi	60 min	2 times	26 weeks	②
Wei Sun 2018 (54)	65.3 ± 4.3	11	12	Simplified 24-Form Tai Chi	60 min	5 times	16 weeks	⑧
Nam-Kuk Son 2016 (53)	71.5 ± 3.6	24	21	Sun-style Tai Chi	60 min	2 times	12 weeks	④⑥⑦⑧
Michel Tousignant 2012 (55)	80.7 ± 6.0	76	76	Conventional Tai Chi	60 min	2 times	15 weeks	⑦

① Fear of Falling (FOF) ②: Falls Efficacy Scale (FES) ③: Time Balance Test (TBT) ④: Timed Up and Go (TUG) ⑤: Functional Reach Distance (FRD) ⑥: maximum walking speed (MWS) ⑦: Berg Balance Scale (BBS) ⑧: One-Leg Standing Test (OLS-C).

### 3.2 Methodological quality assessment of included studie

**Figure 2:**
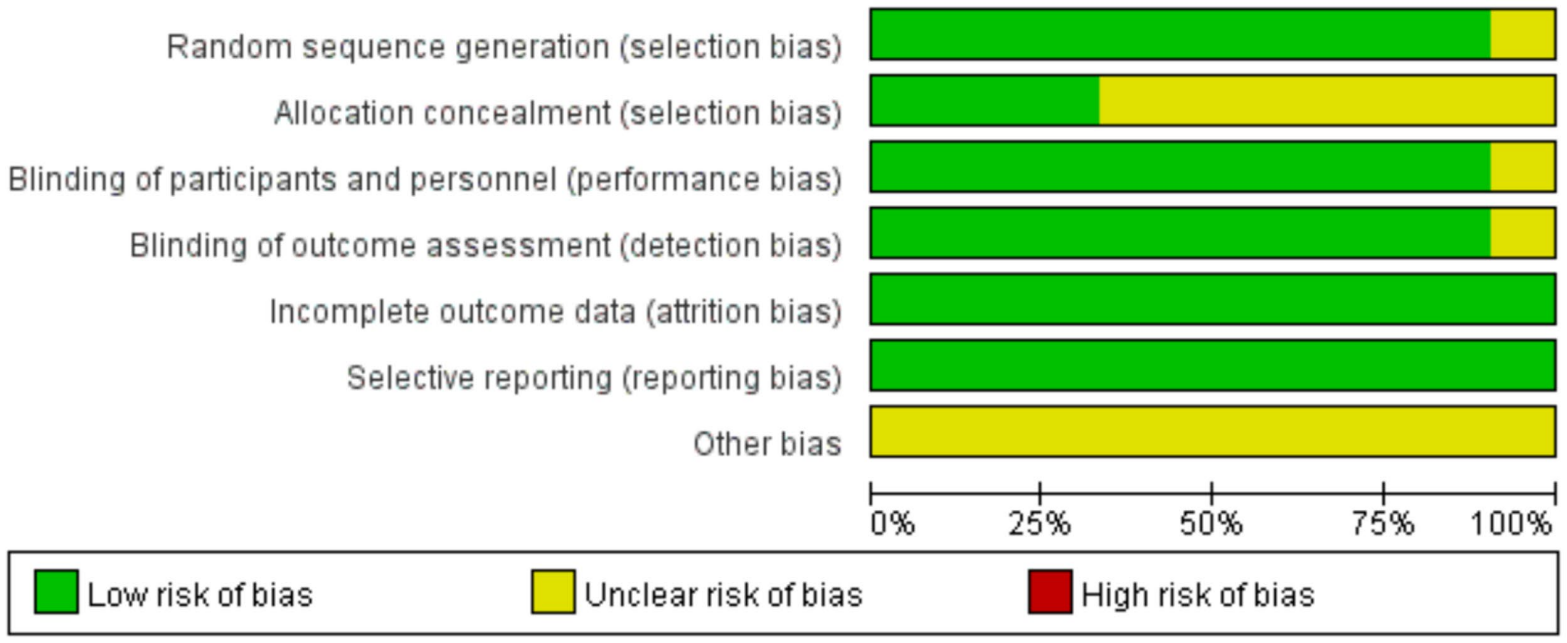
Risk of bias chart.

The methodological quality of the included studies was assessed using the Cochrane Risk of Bias Tool. All 21 included RCTs reported baseline characteristics of the participants, and 19 of them provided a detailed description of the methods used to generate the randomization sequence (17, 20, 40–56), Seven studies provided detailed information on allocation concealment (17, 20, 46, 49, 51, 53, 55), 19 studies reported the implementation of blinding (17, 19, 20, 40, 41, 43–56), Data reporting was complete for all 21 studies, and any missing data or reasons for missing data were thoroughly described. Furthermore, all 21 studies demonstrated selective outcome reporting, and no other sources of bias were identified in these studies. The detailed results of the quality assessment are shown in Figure 2.

### 3.3 Publication Bias

**Figure 3:**
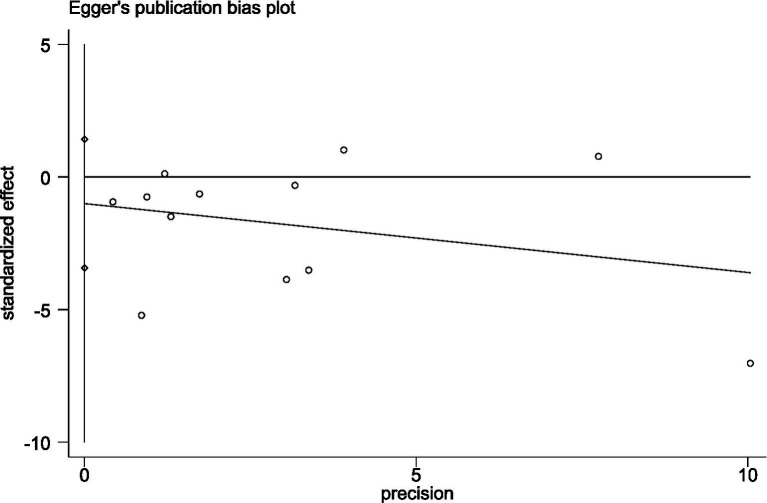
Egger’s test plot for TUG test studies.

**Figure 4:**
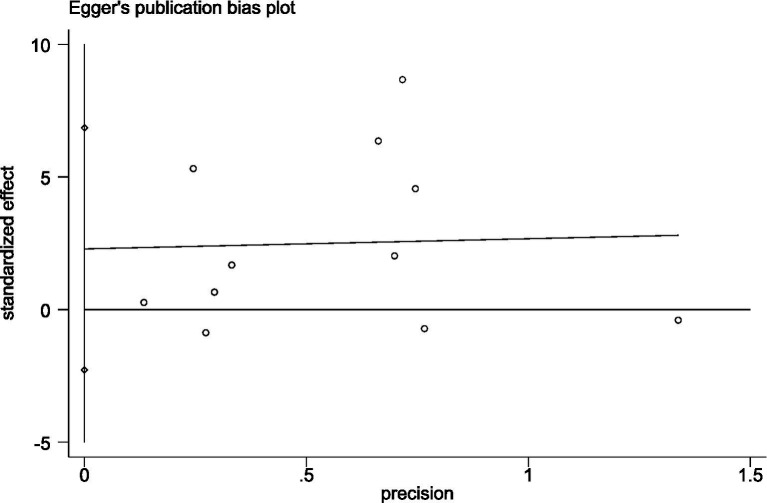
Egger’s test plot for OLS-C test studies.

**Figure 5:**
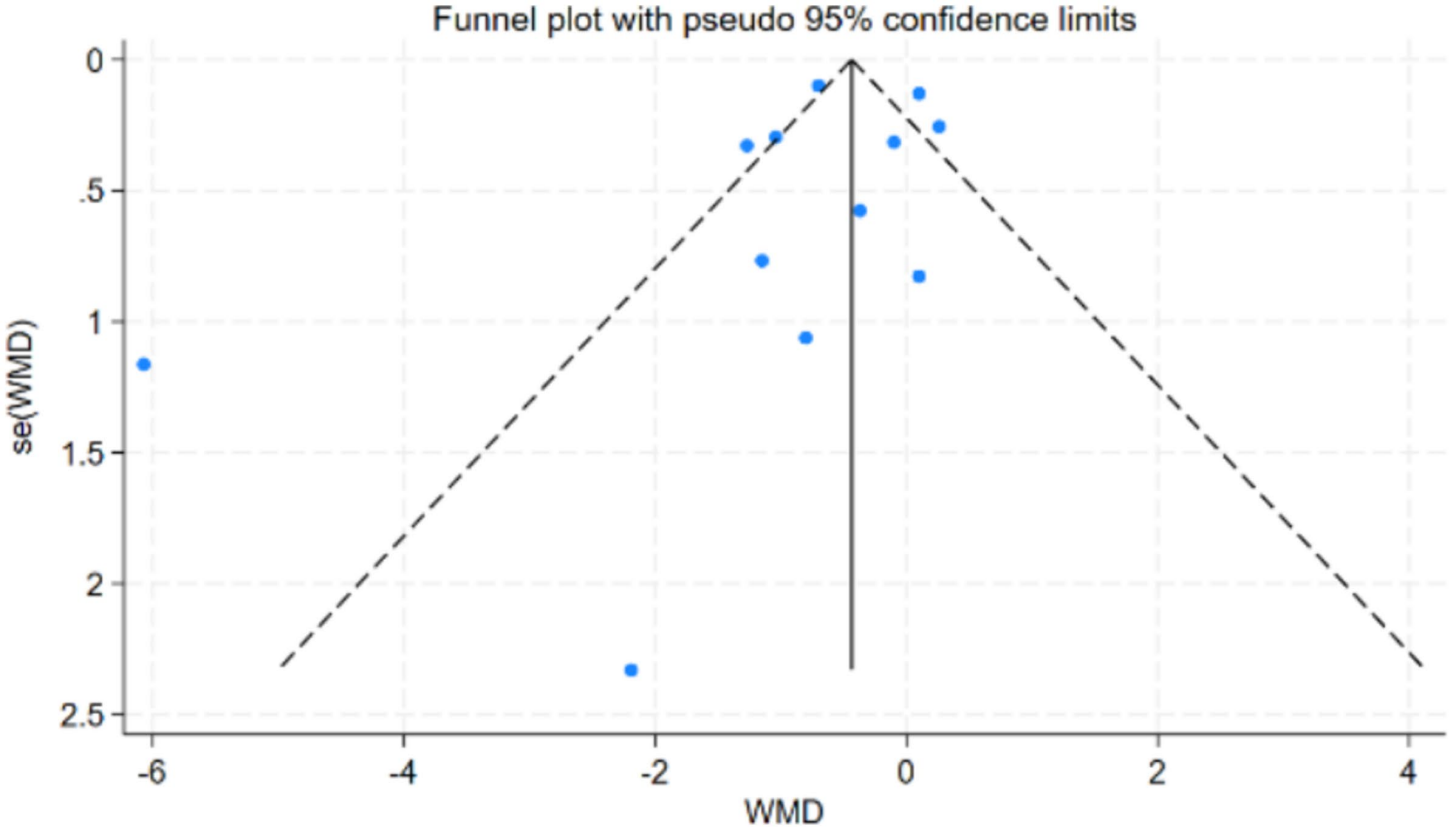
Funnel plot for publication bias in TUG test studies.

**Figure 6:**
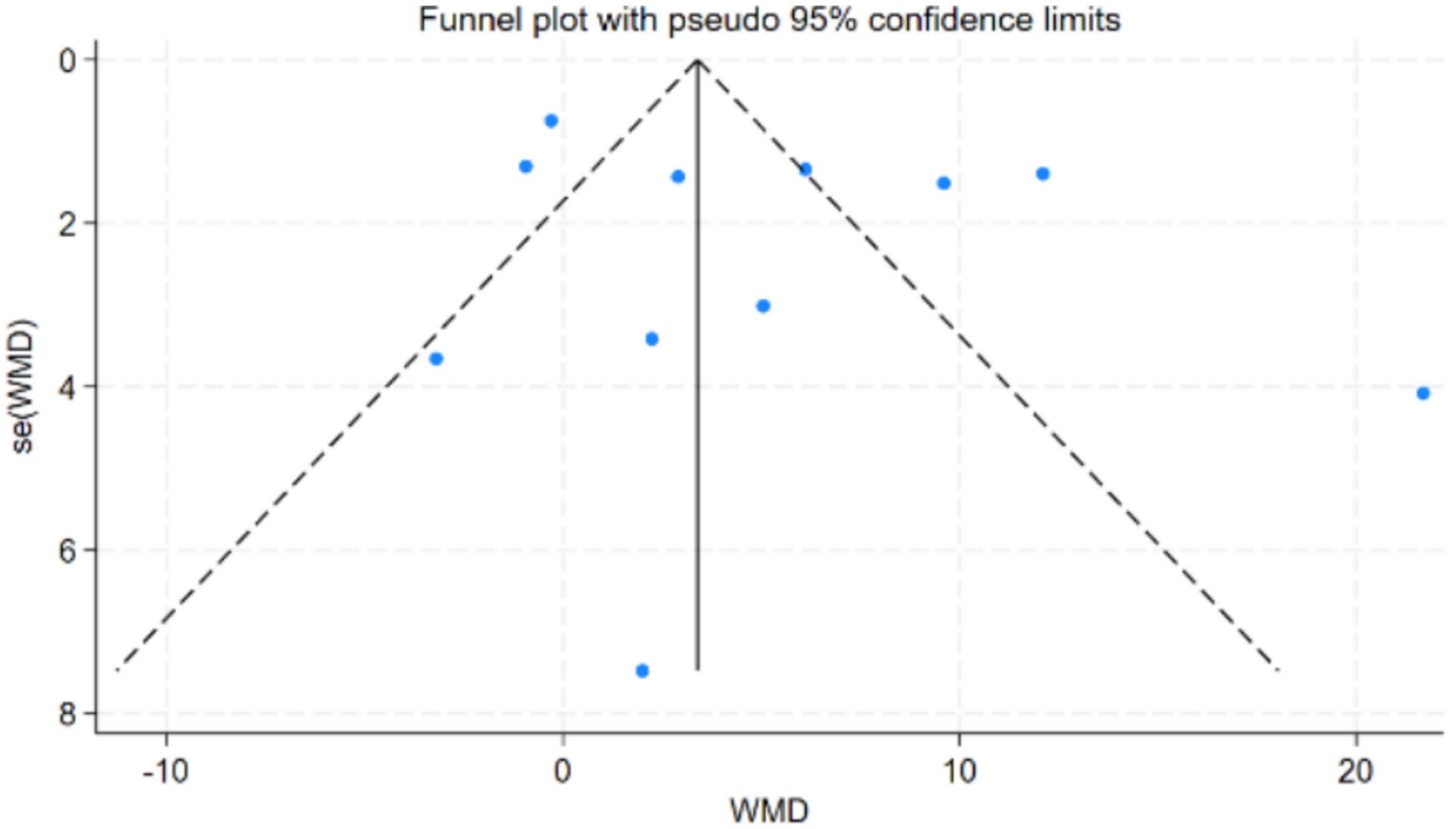
Funnel plot for publication bias in OLS-C test studies.

Publication bias was assessed using Begg’s test and Egger’s test for more than 10 studies involving the Timed Up and Go (TUG) test and the One-Legged Stand (OLS-C) test. In the TUG study results, both Begg’s test (*p* = 0.396) and Egger’s test (*p* = 0.377) reached statistical significance. The funnel plot was nearly symmetrical, indicating that the study results were robust and minimally affected by publication bias. In the OLS-C study results, neither Begg’s test (*p* = 0.86) nor Egger’s test (*p* = 0.286) reached statistical significance, indicating no significant publication bias. Detailed results are shown in Figures 3, 4. The funnel plot was nearly symmetrical, with no apparent small sample effects, suggesting that the overall effect estimate is reliable and minimally influenced by bias. For further details, refer to Figures 5, 6.

### 3.4 Meta-analysis results

#### 3.4.1 Fear of falling

**Figure 7:**
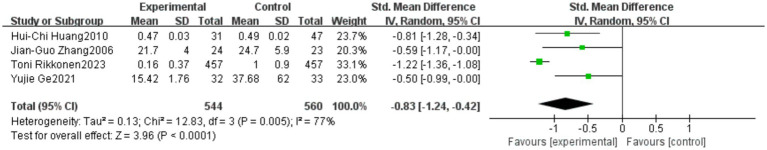
Meta-analysis results of the effect of Tai Chi on fear of falling in the older adults.

A total of four studies reported outcomes related to fear of falling (FOF). The meta-analysis indicated a high level of heterogeneity among these studies (heterogeneity test: χ^2^ = 12.83, *p* = 0.005, *I*^2^ = 77%). Therefore, a random-effects model was applied to calculate the pooled effect size. The standardized mean difference (SMD) was −0.83, with a 95% confidence interval (CI) ranging from −1.24 to −0.42 (*p* < 0.0001). These results suggest that Tai Chi significantly reduces fear of falling in the older adults. Detailed results are presented in Figure 7.

The observed heterogeneity may be attributed to variations in intervention duration, training frequency, baseline age structure of participants, and differences in the instruments used to measure fear of falling (FOF) across the included studies. To assess the robustness of the pooled estimate, a sensitivity analysis was conducted using a leave-one-out method. The results indicated that no single study had a disproportionate influence on the overall effect size, suggesting a relatively stable outcome. Nonetheless, the substantial heterogeneity warrants cautious interpretation of this finding.

#### 3.4.2 Falls Efficacy Scale

**Figure 8:**
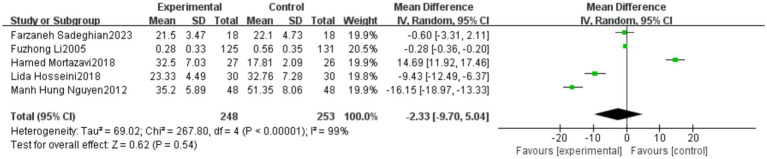
Meta-analysis results of the effect of Tai Chi on Falls Efficacy Scale (FES) in the older adults.

A total of five studies reported outcomes related to falls efficacy (FES). The meta-analysis indicated a high level of heterogeneity among these studies (heterogeneity test: χ^2^ = 267.80, *p* < 0.00001, *I*^2^ = 99%). Therefore, a random-effects model was applied to calculate the pooled effect size. The standardized mean difference (SMD) was −2.33, with a 95% confidence interval (CI) ranging from −9.70 to −5.04 (*p* = 0.54). The overall effect was not statistically significant, suggesting that the current evidence is insufficient to confirm the effectiveness of Tai Chi in improving fall-related self-efficacy in the older adults. Further high-quality studies are needed to validate this conclusion. Detailed results are presented in Figure 8.

The high heterogeneity observed in the FES outcome may be attributed to two primary factors. First, variations in the versions of the Falls Efficacy Scale employed across studies—including differences in language, adaptations, and simplified formats—may have introduced measurement bias, potentially compromising the comparability of results. Second, considerable inconsistencies in intervention protocols, such as training durations ranging from 8 to 26 weeks and the use of different Tai Chi styles, likely contributed to divergent intervention intensities and pacing, thereby affecting the stability of the pooled effect size. Although sensitivity analysis using a leave-one-out approach was conducted to examine the robustness of the findings, the heterogeneity remained substantially high. This suggests that the pooled estimate for this outcome should be interpreted with caution. Future research should prioritize the standardization of both intervention protocols and assessment tools to enhance consistency and comparability across studies.

#### 3.4.3 Time Balance Test

**Figure 9:**
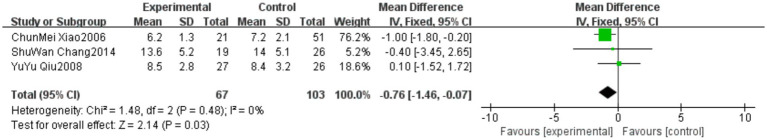
Meta-analysis results of the effects of tai chi on balance ability in older adults.

A total of three studies reported outcomes related to the Timed Balance Test (TBT). The meta-analysis indicated homogeneity among these studies (heterogeneity test: χ^2^ = 1.48, *p* = 0.48, I^2^ = 0%). Therefore, a fixed-effects model was applied to calculate the pooled effect size. The standardized mean difference (SMD) was −0.76, with a 95% confidence interval (CI) ranging from −1.46 to −0.07 (*p* = 0.03). The overall effect was statistically significant, suggesting that Tai Chi has a significant effect on improving balance ability in the older adults. Detailed results are presented in Figure 9.

#### 3.4.4 Timed Up and Go

**Figure 10:**
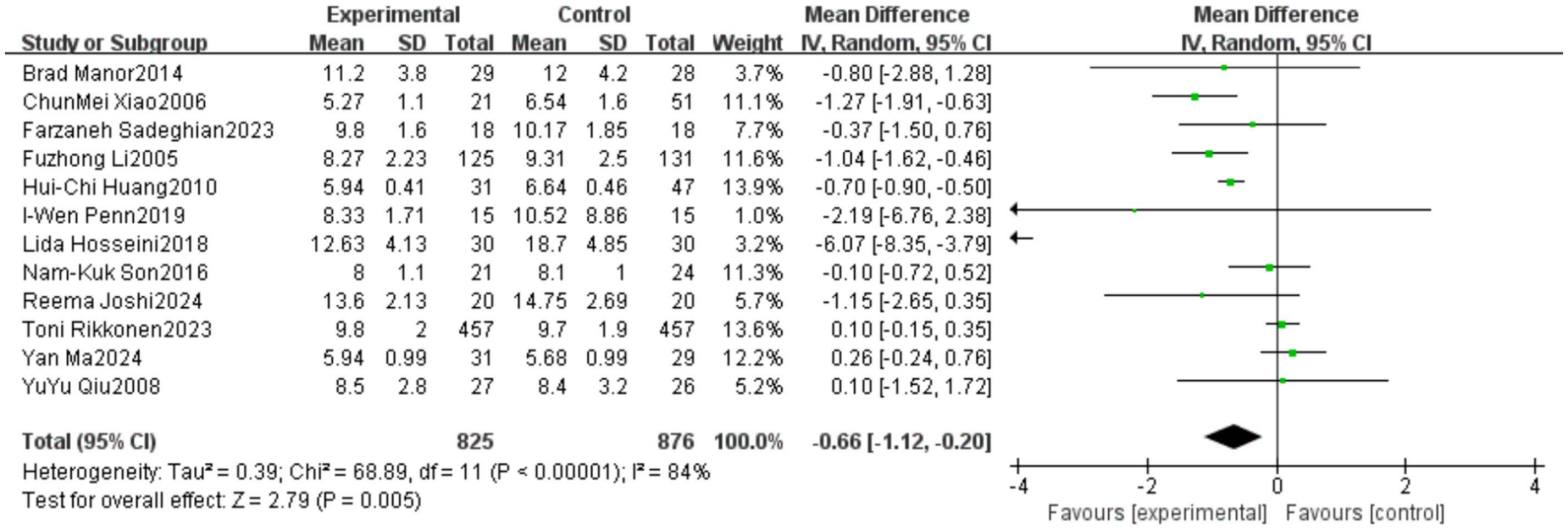
Meta-analysis results of the effect of Tai Chi on the Timed Up and Go (TUG) test in older adults.

A total of 12 studies reported outcomes related to the Timed Up and Go (TUG) test. The meta-analysis indicated a high level of heterogeneity among these studies (heterogeneity test: χ^2^ = 68.89, *p* < 0.00001, *I*^2^ = 84%). Therefore, a random-effects model was applied to calculate the pooled effect size. The standardized mean difference (SMD) was −0.66, with a 95% confidence interval (CI) ranging from −1.12 to −0.20 (*p* = 0.005). The overall effect was statistically significant, suggesting that Tai Chi significantly improves the performance of older adults in the TUG test. Detailed results are presented in Figure 10.

**Figure 11:**
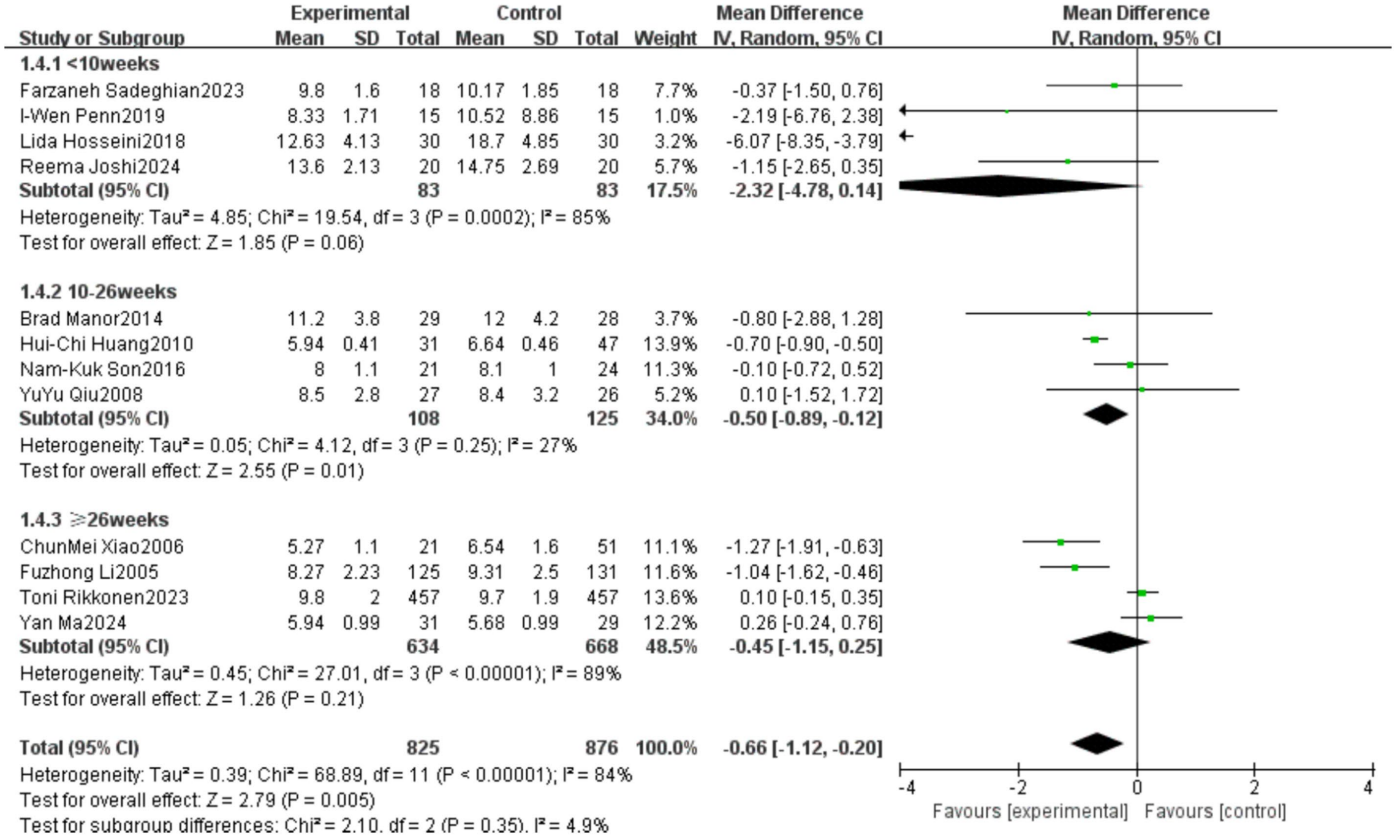
Subgroup analysis of the effect of Tai Chi on the Timed Up and Go (TUG) test in older adults based on total intervention duration.

A subgroup analysis was conducted based on the total duration of Tai Chi intervention, dividing the studies into three subgroups: short-term (<10 weeks), medium-term (10–26 weeks), and long-term (≥26 weeks). The results of the subgroup analysis revealed differences in the effects of Tai Chi on TUG performance among older adults based on intervention duration. In the 10–26 weeks group, the pooled effect size (MD = −0.50, 95% CI [−0.89, −0.12], *p* = 0.01) indicated a statistically significant improvement in TUG performance, demonstrating the effectiveness of Tai Chi in enhancing mobility in older adults. However, in the <10 weeks group (MD = −2.32, 95% CI [−4.78, 0.14], *p* = 0.06) and the ≥26 weeks group (MD = −0.45, 95% CI [−1.15, 0.25], *p* = 0.21), the results did not reach statistical significance. Heterogeneity analysis showed that the 10–26 weeks group exhibited low heterogeneity (*I*^2^ = 27%), whereas the <10 weeks group (*I*^2^ = 85%) and ≥26 weeks group (*I*^2^ = 89%) had high heterogeneity, indicating substantial methodological differences among studies with short-term and long-term interventions. Detailed results are presented in Figure 11.

**Figure 12:**
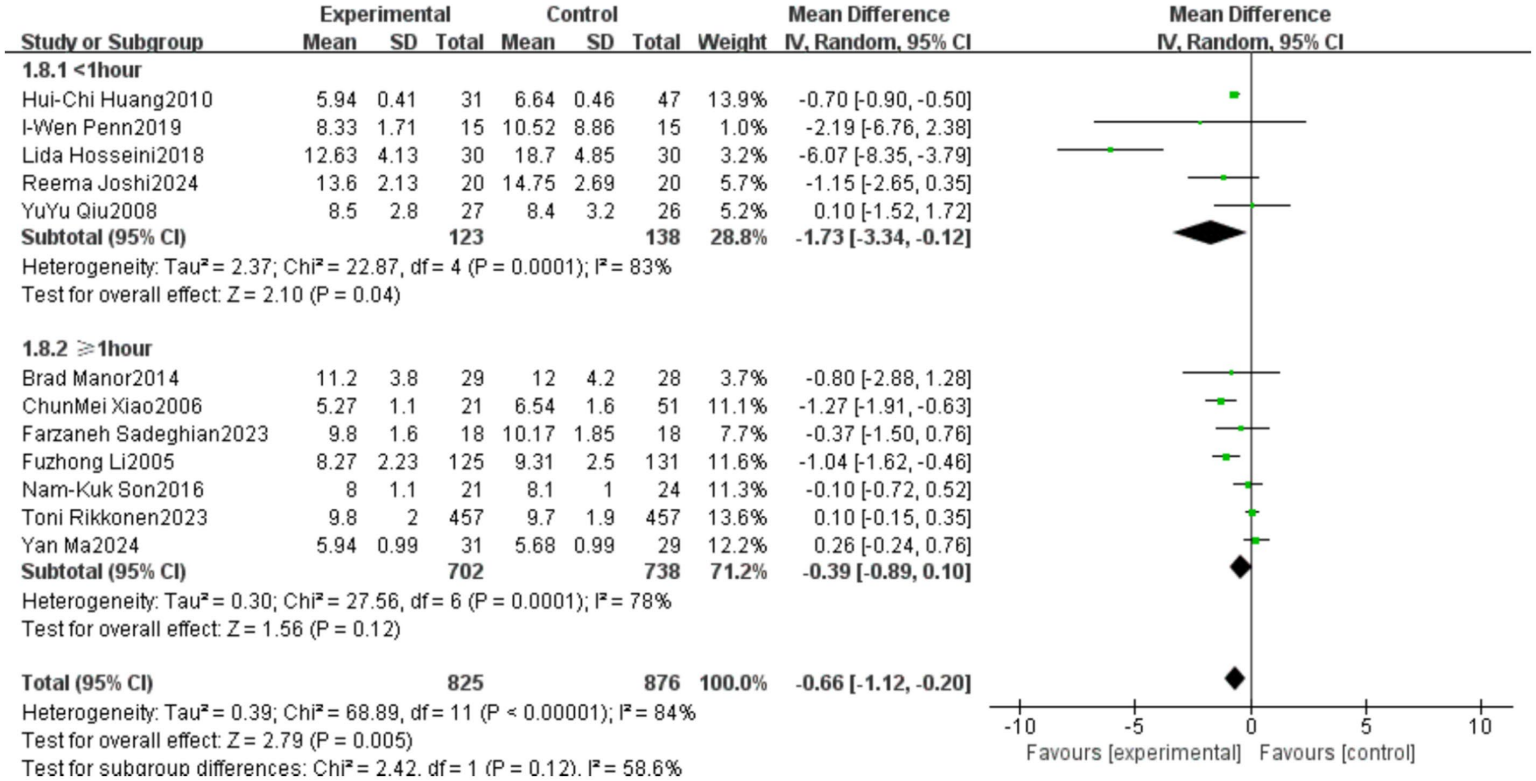
Subgroup analysis of the effect of Tai Chi session duration on the Timed Up and Go (TUG) test in older adults.

The studies were categorized into two subgroups based on the duration of each Tai Chi session: Short duration (<1 h per session), Long duration (≥1 h per session). he subgroup analysis revealed differences in the effects of Tai Chi on TUG performance among older adults based on the duration of each intervention session. In the <1 h per session group, the pooled effect size (MD = −1.73, 95% CI [−3.34, −0.12], *p* = 0.04) indicated a statistically significant improvement in TUG performance, demonstrating the effectiveness of Tai Chi in enhancing mobility in older adults. However, in the ≥1 h per session group (MD = −0.39, 95% CI [−0.89, 0.10], *p* = 0.12), the results did not reach statistical significance. Heterogeneity analysis indicated high heterogeneity in both subgroups, suggesting substantial methodological differences among studies with short-duration and long-duration interventions. Detailed results are presented in Figure 12. Subgroup analysis suggests that variations in intervention duration and protocol design are likely the primary sources of heterogeneity.

#### 3.4.5 Functional reach distance

**Figure 13:**
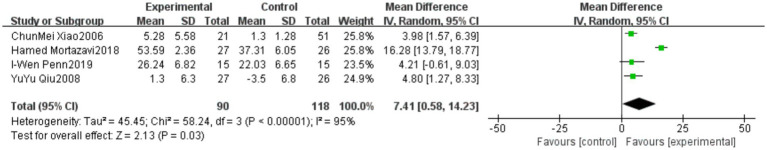
Meta-analysis results of the effect of Tai Chi on functional reach distance (FRD) in older adults.

A total of four studies reported outcomes related to functional reach distance (FRD). The meta-analysis indicated a high level of heterogeneity among these studies (heterogeneity test: χ^2^ = 58.24, *p* < 0.00001, *I*^2^ = 95%). Therefore, a random-effects model was applied to calculate the pooled effect size. The standardized mean difference (SMD) was 7.41, with a 95% confidence interval (CI) ranging from 0.58 to 14.23 (*p* = 0.03). The overall effect was statistically significant, suggesting that Tai Chi significantly improves the performance of older adults in the FRD test. Detailed results are presented in Figure 13. To assess the robustness of the results, a sensitivity analysis was conducted by sequentially excluding individual studies. The results showed that the pooled effect size remained statistically significant (*p* < 0.05), indicating the stability of the findings.

The substantial heterogeneity observed in the FRD outcome (*I*^2^ = 95%) may be primarily attributed to two factors. First, considerable variation existed in the intervention protocols across studies, including differences in training duration (ranging from 8 to 26 weeks), frequency, and intensity, which likely contributed to inconsistencies in treatment effects. Second, discrepancies in outcome assessment procedures and testing environments may have introduced measurement bias. While some studies employed guided or dynamic testing conditions, others relied on self-directed assessments, potentially reducing comparability. Although sensitivity analysis indicated that no single study disproportionately influenced the overall effect size, the high level of heterogeneity suggests that the pooled FRD estimate should be interpreted with caution. Future studies should prioritize standardization of both intervention design and measurement protocols to enhance methodological consistency.

#### 3.4.6 Maximum walking speed

**Figure 14:**
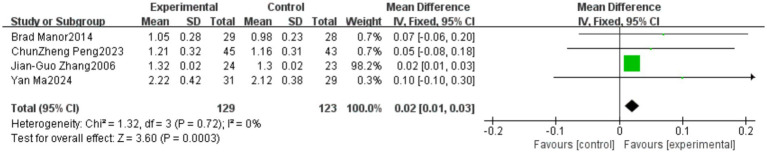
Meta-analysis results of the effect of Tai Chi on maximum walking speed (MWS) in older adults.

A total of four studies reported outcomes related to maximum walking speed (MWS). The meta-analysis indicated homogeneity among these studies (heterogeneity test: χ^2^ = 1.32, *p* = 0.72, *I*^2^ = 0%). Therefore, a fixed-effects model was applied to calculate the pooled effect size. The standardized mean difference (SMD) was 0.02, with a 95% confidence interval (CI) ranging from 0.01 to 0.03 (*p* = 0.003). The overall effect was statistically significant, suggesting that Tai Chi significantly improves the performance of older adults in the MWS test. Detailed results are presented in Figure 14.

#### 3.4.7 Berg Balance Scale

**Figure 15:**
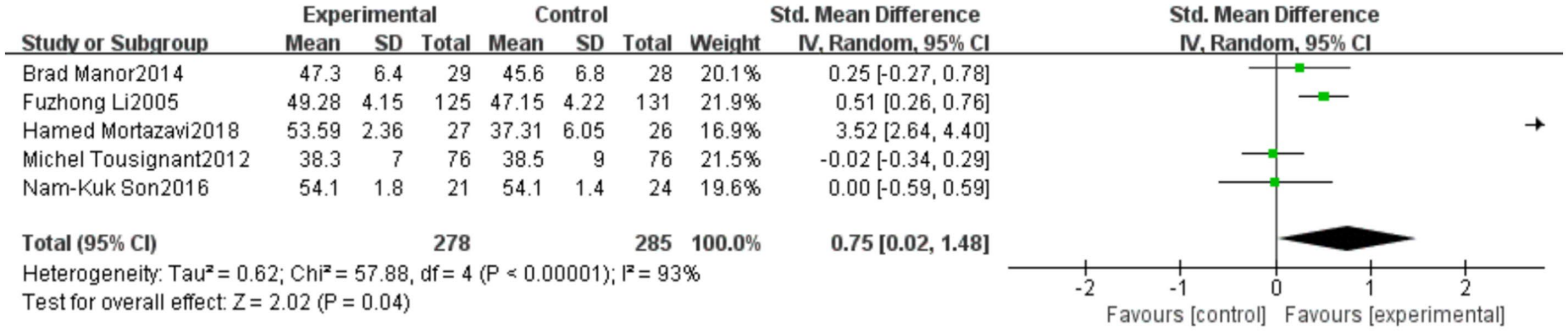
Meta-analysis results of the effect of Tai Chi on Berg Balance Scale (BBS) in older adults.

A total of five studies reported outcomes related to the Berg Balance Scale (BBS). The meta-analysis indicated a high level of heterogeneity among these studies (heterogeneity test: χ^2^ = 57.88, *p* < 0.00001, *I^2^* = 93%). Therefore, a random-effects model was applied to calculate the pooled effect size. The standardized mean difference (SMD) was 0.75, with a 95% confidence interval (CI) ranging from 0.02 to 1.48 (*p* = 0.04). The overall effect was statistically significant, suggesting that Tai Chi significantly improves the performance of older adults in the BBS test. Detailed results are presented in Figure 15. To assess the robustness of the results, a sensitivity analysis was conducted by sequentially excluding individual studies. The results showed that the pooled effect size remained statistically significant (*p* < 0.05), indicating the stability of the findings.

The BBS outcome showed substantial heterogeneity (*I*^2^ = 93%), which may be primarily attributed to three factors. First, considerable variability existed in the types of Tai Chi styles and training intensities applied across studies, leading to inconsistencies in intervention content. Second, the timing of post-intervention assessments varied, with differences in follow-up durations potentially affecting comparability. Third, baseline balance ability differed markedly among study populations, resulting in heterogeneous response magnitudes. Although sensitivity analysis indicated that no individual study exerted a dominant influence on the pooled effect size, the high level of heterogeneity suggests that the findings should be interpreted with caution. Future research should aim to standardize intervention protocols and assessment time points to enhance methodological consistency.

#### 3.4.8 One-Leg Standing Test

**Figure 16:**
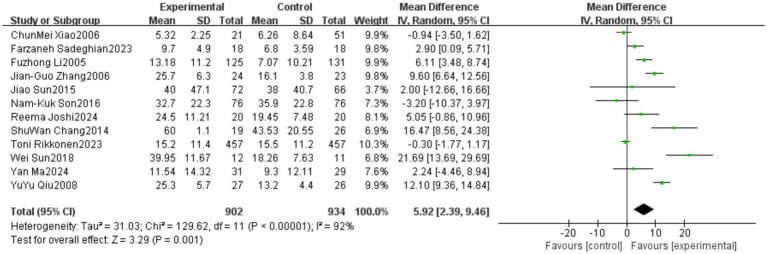
Meta-analysis results of the effect of Tai Chi on one-leg standing duration in older adults.

A total of 12 studies reported outcomes related to the One-Leg Standing Test (OLS-C). The meta-analysis indicated a high level of heterogeneity among these studies (heterogeneity test: χ^2^ = 129.62, *p* < 0.00001, *I*^2^ = 92%). Therefore, a random-effects model was applied to calculate the pooled effect size. The standardized mean difference (SMD) was 5.92, with a 95% confidence interval (CI) ranging from 2.39 to 9.46 (*p* = 0.001). The overall effect was statistically significant, suggesting that Tai Chi significantly improves the performance of older adults in the OLS-C test. Detailed results are presented in Figure 16.

**Figure 17:**
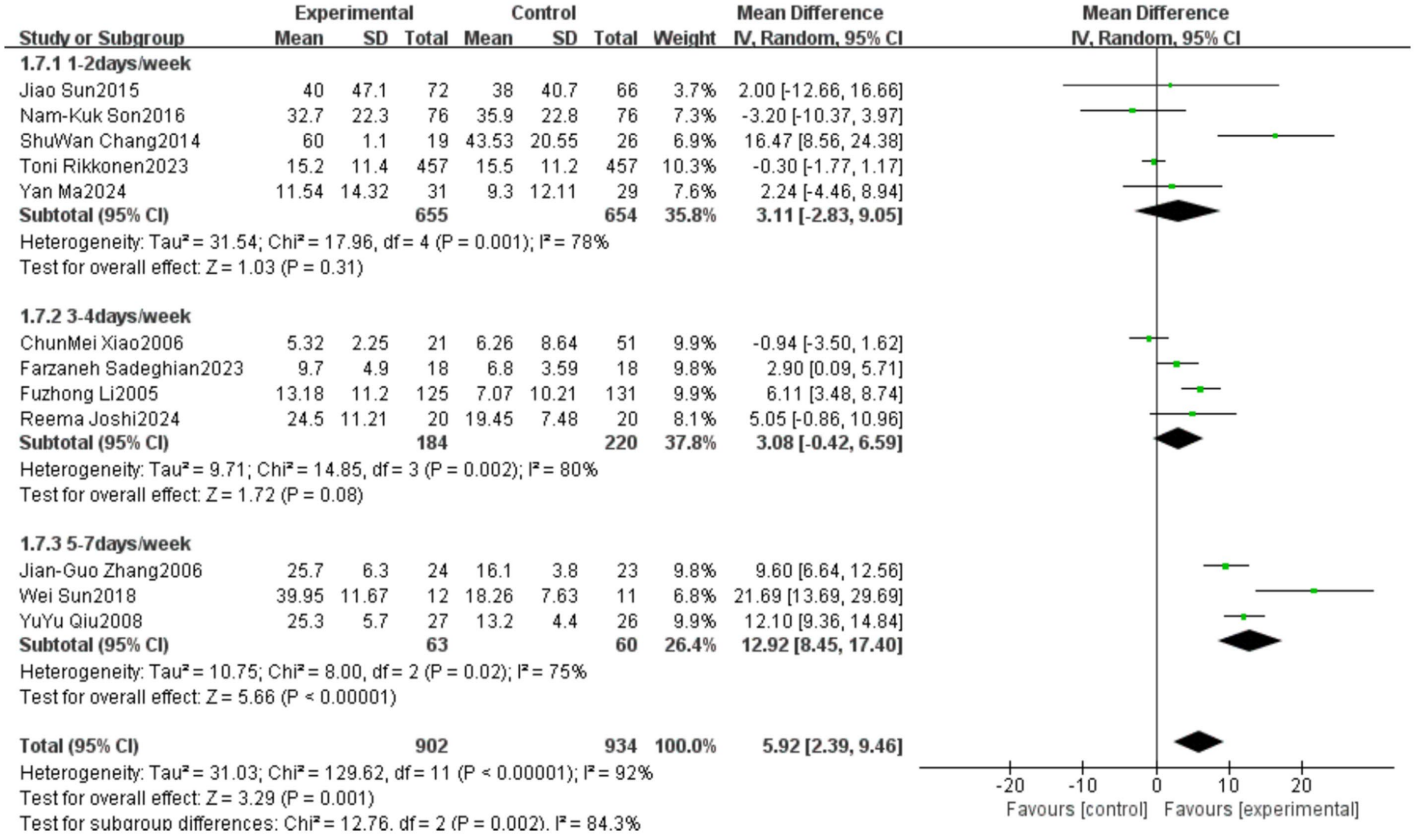
Subgroup analysis of the effect of different Tai Chi intervention frequencies on one-leg standing duration in older adults.

The studies were categorized into three subgroups based on the frequency of Tai Chi intervention: Low frequency (1–2 times per week), Medium frequency (3–4 times per week), High frequency (5–7 times per week). The subgroup analysis results indicated differences in the effects of Tai Chi on one-leg standing performance among older adults based on intervention frequency. In the 5–7 times per week group, the pooled effect size (MD = 12.92, 95% CI [8.45, 17.40], *p* < 0.00001) demonstrated a statistically significant improvement in one-leg standing performance, suggesting that Tai Chi practice at a higher frequency yields substantial benefits. However, in the 1–2 times per week group (MD = 3.11, 95% CI [−2.83, 9.05], *p* = 0.31) and the 3–4 times per week group (MD = 3.08, 95% CI [−0.42, 6.59], *p* = 0.08), although a trend toward improvement was observed, the results did not reach statistical significance. Heterogeneity analysis showed that the I^2^ values were high across all subgroups, indicating substantial methodological differences among the included studies. Detailed results are presented in Figure 17.

**Figure 18:**
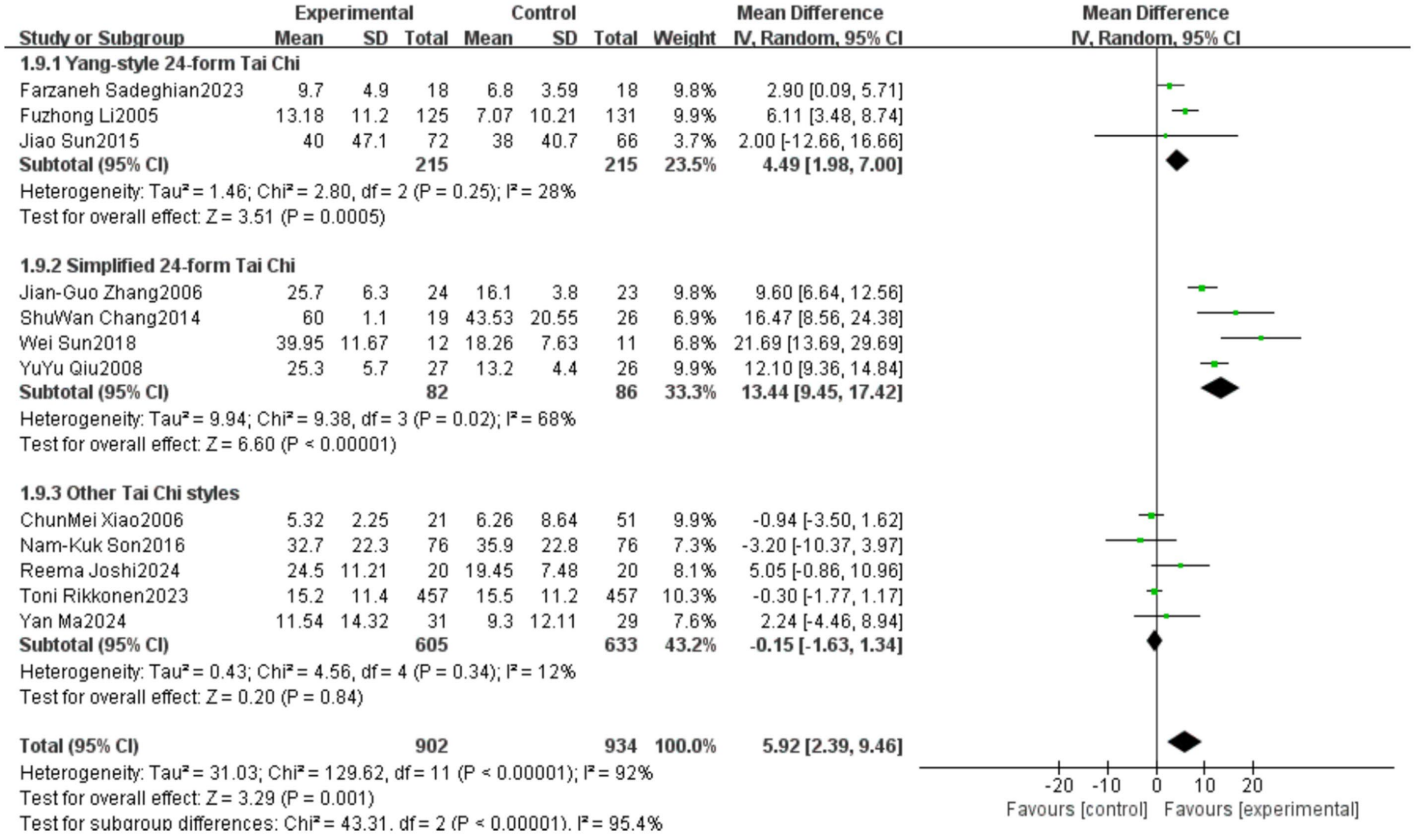
Subgroup analysis of the effect of different Tai Chi styles on one-leg standing duration in older adults.

The studies were categorized into three subgroups based on the Tai Chi style used in the intervention: Yang-style 24-form Tai Chi, Simplified 24-form Tai Chi, Other Tai Chi styles (mixed styles or Sun-style, Chen-style Tai Chi). The subgroup analysis results indicated differences in the effects of various Tai Chi styles on one-leg standing duration in older adults. In the Yang-style 24-form Tai Chi group (MD = 4.49, 95% CI [1.98, 7.00], *p* = 0.0005) and the Simplified 24-form Tai Chi group (MD = 13.44, 95% CI [9.45, 17.42], *p* < 0.00001), the results demonstrated a statistically significant improvement in one-leg standing performance, suggesting that these Tai Chi styles effectively enhance balance in older adults. In the Other Tai Chi styles group, the pooled effect size (MD = −0.15, 95% CI [−1.63, 1.34], *p* = 0.84) indicated that Tai Chi did not significantly improve one-leg standing performance in older adults. Heterogeneity analysis showed that the Yang-style 24-form Tai Chi group (*I*^2^ = 28%) and the Other Tai Chi styles group (*I*^2^ = 12%) had low heterogeneity, suggesting consistent findings across studies. However, the Simplified 24-form Tai Chi group (*I*^2^ = 68%) exhibited high heterogeneity, indicating substantial variation in study results. Detailed results are presented in Figure 18. Subgroup analysis indicates that the use of different Tai Chi styles is a major contributor to the high heterogeneity observed in the OLS-C outcome.

### 3.5 GRADE assessment of outcome evidence

In this study, we employed the GRADE (Grading of Recommendations, Assessment, Development, and Evaluation) framework to assess the quality of evidence for each outcome. Based on the assessment, the quality of evidence for each outcome was rated as either high or moderate. Several factors were considered in this evaluation, including the risk of bias in the study designs, inconsistency of results across studies, imprecision due to small sample sizes, as well as concerns regarding indirectness or publication bias.

Fear of Falling (FOF) and maximum walking speed (MWS) were rated as high-quality evidence due to consistent and reliable results from multiple high-quality randomized controlled trials (RCTs).

Time Balance Test (TBT) and Berg Balance Scale (BBS) also showed significant effects but were rated as moderate-quality evidence due to the heterogeneity observed across studies.

Other outcomes such as Falls Efficacy Scale (FES), Timed Up and Go (TUG), One Leg Stand (OLS-C), and functional reach distance (FRD) were rated as moderate quality. This was primarily due to the high heterogeneity between studies and relatively small effect sizes for some outcomes. The detailed descriptions and the GRADE assessment results for each outcome are summarized in the table, providing a comprehensive foundation for the reliability and validity of the study’s conclusions. Future research should aim to address the potential sources of heterogeneity and enhance the consistency of study designs to improve the overall quality of evidence (please refer to Table 4).

**Table 4:** Summary of findings and GRADE assessment for all outcomes.

Outcome	Illustrative comparative risks (95% CI) corresponding riskwith exercise	Number of participants (studies)	Certainty of the evidence
FOF	The mean FOF score post-intervention was 0.83 points lower (−1.24 to −0.42) compared to usual exercise.	775 (4 studies)	High quality
FES	The mean FES score post-intervention was 2.33 points lower (−9.70 to −5.04) compared to usual exercise.	961 (5 studies)	Moderate quality ^‡+†^
TBT	The mean TBT score post-intervention was 0.76 points higher (−1.46 to −0.07) compared to usual exercise.	190 (3 studies)	High quality
TUG	The mean TUG score post-intervention was 0.66 points lower (−1.12 to −0.20) compared to usual exercise.	592 (12 studies)	Moderate quality^+†^
OLS-C	The mean OLS-C score post-intervention was 5.92 points higher (2.39–9.46) compared to usual exercise.	961 (12 studies)	Moderate quality^+†^
FRD	The mean FRD score post-intervention was 7.41 points higher (0.58–14.23) compared to usual exercise.	868 (4 studies)	Moderate quality^+†^
MWS	The mean MWS post-intervention was 0.02 meters per second higher (0.01–0.03) compared to usual exercise.	2,408 (4 studies)	High quality
BBS	The mean BBS score post-intervention was 0.75 points higher (0.02–1.48) compared to usual exercise.	868 (5 studies)	Moderate quality^‡+†^

High quality: Further research is very unlikely to change our confidence in the estimate of effect. Moderate quality: Further research is likely to have an important impact on our confidence in the estimate of effect and may change the estimate. Low quality: Further research is very likely to have a significant impact on our confidence in the estimate of effect and is likely to change the estimate. Very low quality: We have a high degree of uncertainty about the estimate. +: Heterogeneity. ‡: Imprecision. †: Publication bias.

## 4 Discussion

This systematic review and meta-analysis included a total of 21 studies, encompassing 2,408 healthy older adults, aiming to investigate the effects of Tai Chi on improving balance ability and reducing fall risk in the older adults. The results of this study indicate that Tai Chi significantly improves balance ability in healthy older adults. Specifically, Tai Chi practice led to a significant increase in Berg Balance Scale (BBS) scores, a notable reduction in Timed Up and Go (TUG) test duration, a significant extension of one-leg standing time (OLS-C), and a marked improvement in functional reach distance (FRD). These findings highlight the positive effects of Tai Chi on balance enhancement in the older adults. Furthermore, Tai Chi was also associated with significant improvements in fear of falling (FOF) scores, performance in the Timed Balance Test (TBT), and maximum walking speed (MWS), demonstrating its potential in reducing fall risk among healthy older adults. However, the results for the Falls Efficacy Scale (FES) did not reach statistical significance, suggesting that the effects of Tai Chi may vary depending on the specific balance and functional assessment tools used.

### 4.1 Sources of heterogeneity and methodological considerations

This meta-analysis revealed varying levels of heterogeneity across different outcome measures, reflecting substantial differences in intervention designs, measurement tools, and sample characteristics across the included studies.

To further address the observed high heterogeneity in several outcome measures, we conducted an in-depth exploration of potential sources related to variations in intervention implementation details. Specifically, differences in Tai Chi protocols—such as style (e.g., Yang-style, 24-form simplified), session frequency, session duration, and total intervention length—are considered major contributors to between-study variability. Additionally, inconsistencies in methodological reporting, outcome measurement tools, and population characteristics (e.g., age range, baseline balance ability) further compounded heterogeneity. Recognizing these factors, we recommend that future studies adhere to standardized intervention frameworks and detailed reporting practices to enhance the interpretability and comparability of findings in meta-analyses.

For outcomes without effective subgroup analyses or limited heterogeneity control, such as the Falls Efficacy Scale (FES), functional reach distance (FRD), and Berg Balance Scale (BBS), high heterogeneity was observed (*I*^2^ = 99, 95, and 93%, respectively). The major sources of heterogeneity for these outcomes include: 1. significant variations in intervention protocols, with training durations ranging from 8 to 26 weeks, differing frequencies, session durations, and Tai Chi styles; 2.differences in measurement tools and methods, such as variations in the versions of the FES scale and its translations, different methods of assessing FRD, and variations in the timing and procedures for BBS testing; 3.baseline differences in the study populations, such as variations in initial balance ability and individual responses to interventions. Although sensitivity analysis showed no single study exerting disproportionate influence on the pooled effect, the substantial heterogeneity in these outcomes indicates the need for greater standardization of intervention designs and measurement protocols in future studies to enhance the reliability and interpretability of meta-analytic findings.

For outcomes with subgroup analyses, the sources of heterogeneity were more clearly defined. In the Timed Up and Go (TUG) test, subgroup analysis revealed that studies with an intervention duration of 10–26 weeks demonstrated significant effects with reduced heterogeneity (*I*^2^ = 27%), while shorter (<10 weeks) and longer (≥26 weeks) interventions showed higher heterogeneity and nonsignificant effects. These results suggest that insufficient intervention duration may not allow for sufficient physiological adaptation, while prolonged interventions may suffer from reduced adherence or other external factors, compromising consistency in treatment effects.

In the One-Leg Standing with Eyes Closed (OLS-C) outcome, significant heterogeneity (*I*^2^ = 92%) was observed, with subgroup analysis indicating that Tai Chi style was a major contributor to this variability. Studies using the simplified 24-form Tai Chi showed higher heterogeneity (*I*^2^ = 68%), while those using the Yang-style Tai Chi or other styles exhibited relatively lower heterogeneity. This highlights the importance of standardizing Tai Chi training content to ensure greater consistency in intervention effects.

In summary, while the overall findings support the positive effects of Tai Chi on improving balance and reducing fall risk in healthy older adults, the high heterogeneity observed in several outcomes, including FES, TUG, and OLS-C, suggests that the results should be interpreted with caution. Future research should focus on standardizing intervention duration, frequency, Tai Chi style selection, and outcome measurement protocols to improve the comparability and reliability of findings.

### 4.2 The impact of Tai Chi on balance ability in older adults

This study evaluated the effects of Tai Chi on balance ability in older adults using the Timed Up and Go (TUG) test, One-Leg Standing Time (OLS-C), Berg Balance Scale (BBS), and functional reach distance (FRD) test. The key findings are as follows: The conclusions drawn from the Timed Up and Go (TUG) test are consistent with the findings of Mao et al. (57), The latter also reported that Tai Chi intervention significantly improved the Timed Up and Go (TUG) test performance in healthy older adults, further confirming the crucial role of Tai Chi in enhancing balance ability in the older adults. In the One-Leg Standing Time (OLS-C) test, the Tai Chi group demonstrated significantly better performance than the control group, indicating that Tai Chi effectively prolonged OLS-C duration in healthy older adults, This finding differs from the results reported by Song et al. (27), That study indicated that while Tai Chi significantly improves dynamic balance ability in older adults, its effects on static balance tests, such as One-Leg Standing Time (OLS-C), were relatively limited. The potential reason for this discrepancy is that the present study effectively controlled heterogeneity through subgroup analysis and sensitivity analysis, providing a more precise understanding of the intervention effects. In contrast, the study by Song et al. did not perform a subgroup analysis, which may have limited their ability to fully explore the variations in intervention effects across different populations, potentially leading to differing conclusions; In the Berg Balance Scale (BBS) test, Tai Chi significantly improved BBS scores in healthy older adults, This result is not consistent with the findings of Zhang et al. (58), That study suggested that the effect of Tai Chi on improving Berg Balance Scale (BBS) scores in older adults was inconclusive. This discrepancy may stem from the greater heterogeneity of participants in Zhang et al.’s study, where variations in health status could have led to higher result variability. In contrast, our study included a more homogeneous sample, reducing such variability and yielding more consistent findings; In the functional reach distance (FRD) test, Tai Chi significantly increased the functional reach distance in healthy older adults, This finding is consistent with the results of Bertolini et al. (59), With the improvement observed in our study being even more pronounced.

### 4.3 The effects of Tai Chi on fall prevention in older adults

This study explored the effects of Tai Chi on fear of falling (FOF) in older adults. The results showed that the Tai Chi group performed significantly better in FOF assessments compared to the control group, This finding is consistent with the conclusions of Zhang (58) and Ge et al. (44), They suggested that Tai Chi helps balance vital energy (Qi) and blood circulation, promotes metabolism, and possesses anti-aging effects. Long-term practice can significantly enhance an individual’s immune function and body control, thereby contributing to fall prevention and alleviating fear of falling. In the Timed Balance Test (TBT), Tai Chi significantly improved the performance of healthy older adults, This finding is not consistent with the results of Li et al. (25), The results of the latter study indicated that although the Tai Chi group showed some improvement in the Timed Balance Test (TBT) compared to the control group, the difference did not reach statistical significance. In the maximum walking speed (MWS) test, Tai Chi was found to significantly enhance the maximum walking speed in healthy older adults, This finding is consistent with the results of Meng et al. (60), The latter also reported that Tai Chi intervention significantly improved the maximum walking speed (MWS) in healthy older adults. This finding further confirms the crucial role of Tai Chi in reducing fall risk among the older adults. In the Falls Efficacy Scale (FES) test, although the experimental group exhibited lower FES scores, suggesting that Tai Chi may effectively reduce fall risk and improve fall efficacy, the confidence interval crossed zero, and the difference did not reach statistical significance. The current analytical evidence is insufficient to confirm the effectiveness of Tai Chi, possibly due to the high heterogeneity among studies. Therefore, these findings should be interpreted with caution. To further validate the effects of Tai Chi, future research should focus on more refined subgroup analyses and high-quality randomized controlled trials.

### 4.4 The optimal dose of tai chi exercise for healthy older adults

#### 4.4.1 Duration of continuous intervention

This study indicates that the effects of Tai Chi on balance ability in older adults vary significantly depending on the duration of continuous intervention. Specifically, mid-term intervention (10–26 weeks) demonstrated a significant improvement in the Timed Up and Go (TUG) test among older adults, whereas short-term interventions (<10 weeks) and long-term interventions (≥26 weeks) did not achieve statistically significant training effects. This may be because short-term interventions (<10 weeks) are insufficient to achieve significant training effects, while long-term interventions (≥26 weeks) may be influenced by factors such as decreased adherence or variations in training intensity, potentially reducing the intervention’s effectiveness, This finding is similar to the results reported by Wang et al. (61). The short-term and long-term intervention groups exhibited high heterogeneity (*I*^2^ = 85% and *I*^2^ = 89%, respectively), which may be influenced by factors such as intervention methods and participant characteristics. This suggests that future studies should further standardize intervention protocols to enhance comparability. Overall, a Tai Chi intervention lasting 10–26 weeks may represent the optimal time window for enhancing balance ability and reducing fall risk in older adults. However, further high-quality randomized controlled trials are needed to confirm these findings.

#### 4.4.2 Weekly intervention frequency

The World Health Organization (WHO) recommends that older adults engage in at least three sessions of moderate-intensity physical activity per week to promote bone health, improve muscle strength, and maintain overall physical function, thereby effectively reducing the risk of falls (62). Multiple randomized controlled trials have demonstrated that engaging in Tai Chi training at least three times per week can significantly improve balance ability in older adults and effectively reduce the risk of falls (63–65). These conclusions were further confirmed in our study, which explored the intervention effects of different training frequencies. Our findings indicate that Tai Chi interventions performed 5–7 times per week resulted in significant improvements in one-leg standing balance (OLS-C) among older adults, whereas training frequencies of 1–2 times per week and 3–4 times per week did not achieve statistically significant effects. However, all three subgroups exhibited high heterogeneity, which may be influenced by variations in training protocols, differences in exercise environments, and discrepancies in monitoring and recording standards. Overall, a Tai Chi intervention performed 5–7 times per week may represent the optimal training frequency for enhancing balance ability and reducing fall risk in older adults. Future research should comprehensively investigate the variability of training protocols, differences in exercise environments, and discrepancies in monitoring and recording standards. Additionally, both qualitative and quantitative aspects of Tai Chi programs should be considered to ensure the safety and effectiveness of the intervention, thereby optimizing its potential health benefits.

#### 4.4.3 Duration of each intervention session

This meta-analysis examined the effects of different session durations of Tai Chi interventions on balance ability in older adults. The results indicated that Tai Chi sessions lasting less than 1 h per session were associated with more significant improvements in the Timed Up and Go (TUG) test, whereas interventions lasting ≥1 h per session did not show statistically significant improvements. This discrepancy may be attributed to higher adherence rates in the <1-h group. However, both subgroups exhibited high heterogeneity (*I*^2^ = 83% and *I*^2^ = 78%, respectively). Future research should focus on standardizing intervention protocols to enhance the generalizability and clinical applicability of Tai Chi training programs.

#### 4.4.4 Tai Chi styles

This study indicates that different Tai Chi styles have significantly varying effects on balance ability in older adults. The intervention groups practicing Yang-style 24-form Tai Chi and Simplified 24-form Tai Chi showed significant improvements in the One-Leg Standing Time (OLS-C) test. In contrast, intervention groups practicing other Tai Chi styles did not achieve statistically significant training effects. This finding is similar to the results reported by Cui (23) and Wang et al. (66). Zou et al. (67) suggested that the Yang-style 24-form Tai Chi emphasizes continuity and fluidity of movements, characterized by gentle, slow, and expansive postures. It focuses on maintaining an upright and stable body posture while promoting relaxation and natural body movements. These characteristics positively influence balance parameters and musculoskeletal flexibility in older adults, making it a suitable practice for individuals across different age groups within the older adults. Liang et al. (68) suggested that the Simplified 24-form Tai Chi is a standardized routine derived from Yang-style Tai Chi. It features smooth movements, even pacing, and a well-structured sequence, making it particularly suitable for older adults. This style has been shown to significantly enhance balance ability in the older adults.

The Yang-style 24-form Tai Chi group and other Tai Chi style groups exhibited relatively low heterogeneity (*I*^2^ = 28% and *I*^2^ = 12%, respectively). In contrast, the Simplified 24-form Tai Chi group showed higher heterogeneity (*I*^2^ = 68%), which may be influenced by factors such as intervention methods and participant characteristics. This suggests that future studies should further standardize intervention protocols to enhance comparability. Overall, Yang-style 24-form Tai Chi and Simplified 24-form Tai Chi appear to be the optimal choices for enhancing balance ability and reducing fall risk in older adults. However, further high-quality randomized controlled trials are needed to validate these findings.

## 5 Limitations

Several limitations should be considered when interpreting the results of this systematic review and meta-analysis. First, although a comprehensive search strategy was employed, hand-searching of grey literature and reference lists was not conducted, which may have led to the omission of relevant studies. Second, the included studies exhibited considerable heterogeneity in terms of intervention duration, Tai Chi style, frequency, and outcome measures, potentially affecting the consistency of the pooled results. Third, the majority of the included studies were conducted in Asian countries, which may limit the generalizability of the findings to other populations and cultural contexts. Fourth, due to the lack of individual participant data, subgroup analyses based on age, sex, or comorbidity were not feasible. Finally, some studies had small sample sizes or methodological limitations, such as unclear allocation concealment or lack of blinding, which may have introduced bias and impacted the overall quality of evidence.

## 6 Conclusion

This study conducted a quantitative analysis of the effects of Tai Chi on balance ability and fall risk reduction in healthy older adults, while also updating the existing literature. The results indicate that Tai Chi significantly enhances balance ability and reduces fall risk in healthy older adults, particularly when the training duration does not exceed 1 h per session, the training frequency exceeds 3 times per week, and the intervention period lasts between 10 and 26 weeks. Additionally, different Tai Chi styles exhibit varying effects on enhancing balance ability and reducing fall risk in healthy older adults, with Simplified 24-form Tai Chi and Yang-style 24-form Tai Chi demonstrating superior effectiveness compared to other styles. The findings of this study are applicable to healthy older adults aged 60 and above; however, further research is needed to validate their applicability in other populations.

While this meta-analysis has focused on the effects of Tai Chi on balance and fall risk, it is also important to view Tai Chi as a multifaceted practice that offers benefits beyond just the physical domain. Tai Chi can be understood as involving three key elements: first, slow and attentive movement, which facilitates the balance between attentional engagement and attentional restoration; second, gentle physical exercise, which supports postural control and stability; and third, the shared practice aspect, which fosters a sense of social connection and community. These elements contribute to Tai Chi’s effectiveness in maintaining balance in older adults, not only through physiological mechanisms but also by addressing cognitive and social needs.

This conceptual framework aligns with recent theories of attentional engagement and restoration, such as the work of Schumann et al. which suggests that slow, continuous attention can stabilize cognitive resources and enhance motor control during sustained movement (28). Furthermore, the distinction between tiredness and relaxation, as discussed by Steghaus and Poth, provides a useful lens through which to understand the low-arousal movement states inherent in Tai Chi (29). These states, characterized by a balance between physical relaxation and mental focus, may contribute to improved mood and readiness for physical activity, as outlined in Thayer’s biopsychology of mood and arousal (30).

Incorporating introspective measures into future studies on Tai Chi could help deepen our understanding of these low-arousal states and their relationship to both physiological activation and psychological well-being. This would provide a more nuanced understanding of how Tai Chi’s unique combination of physical, cognitive, and social components contributes to its efficacy in enhancing balance and preventing falls in older adults.

## Data Availability

The original contributions presented in the study are included in the article/Supplementary material, further inquiries can be directed to the corresponding author.
